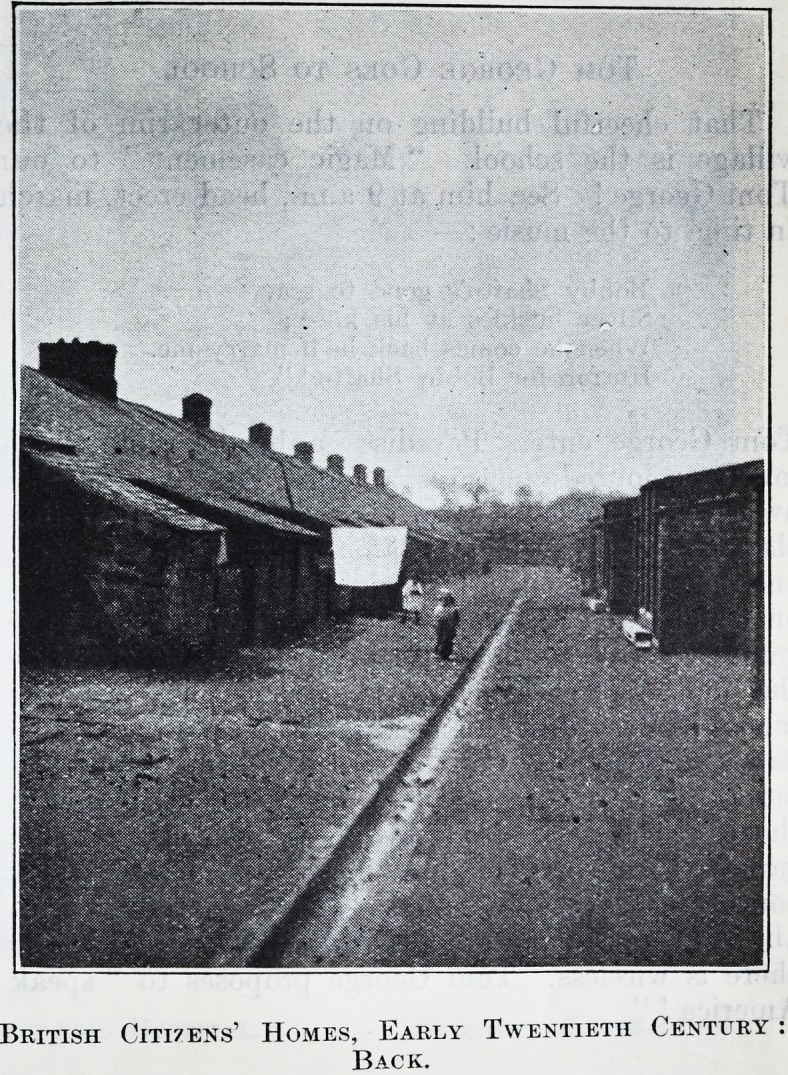# Tom George

**Published:** 1924-07

**Authors:** H. S. Cooper Hodgson


### TOM GEORGE: A STUDY.

By MISS H. S. COOPER HODGSON

Tom George has an odd kind of picture gallery. All the houses in his street have lean-to pantries, with tiny windows, the upper half glass, the lower latticed wood. It is traditional to keep the family egg-cups on the ledge formed by the wooden bottom of the glass section. Every variety of egg-cup is here displayed—white, red, and blue china, and metal. Occasionally other small, gay articles appear, a teapot of Japanese design, or one shaped like a tomato. Sometimes the mite of a window has a blind and lace curtains duly tied with ribbon. A royal blue egg-cup or a brave scarlet tomato teapot gives welcome colour to Tom George's drab world. There are no gardens, the front door opening directly into the street. Front and back streets are unmade, seas of mud in winter, source of dust storms in summer. The front view is another row of drab houses, or, perchance, a pit " stack " or heap ; the back view a row of closets and ashpits, standing out, naked and unashamed, down the middle of the street.

British Citizens' Homes, Early Twentieth Century: Front

British Citizens' Homes, Early Twentieth Century: Back.

### " Houses " at £15 Each.

Thirty years ago all the houses in Tom George's street changed hands at £15 a-piece. And dear at the price ! They have one room up and one room down, a lean-to pantry and scullery ; the staircase goes up out of the living-room (the family's boots are kept here, a pair at each step side), and the front door opens straight into it. Tom George's father, mother, and baby sister sleep in the big bed downstairs, and there is a turn-up bed for the girls ; the rest of the family lie upstairs. When Tom George had measles his mother kept him downstairs, there being no fireplace above ; he got broncho-pneumonia and nearly died. No wonder, with cooking and washing going on, and the door opening straight on to the bed every half-hour or so, in a time of bitter winter gales. As his mother says, " You ought to have heard the doctor and the district nurse ! They did go on proper about the housing ! " When he got well he was sent out to play with Polly. There was nowhere to play except the unmade street, water standing in pools everywhere. So they splashed about a bit until mother worriedly hauled them back to the overcrowded living-room.

### No Place for Children.

Tom George and Polly have exciting times in their street, notably fortnightly, when the men come round to empty the closets, depositing the refuse in heaps pending its removal by carts. Tom George and Polly are much interested in these heaps and scuttle happily about them in the absence of the eye of authority, maternal or scavengerial, for though Tom George's village is emphatically no place to bring up children in, every man there is a child's guardian. They can be seen about the level-crossings and the mineral railways, patiently shooing small fry out of danger with laughing admonition, " Get away home to th' mammy ! " The children are bright as buttons. Polly's mother is washing and falls short of soap. She is tired with

"hugging" water from the street stand-pipe. (There are no wash-houses, or indoor water supply or sink. Oh dear no!) So Polly, aged three, is sent to the shop, and stumps complacently off in a home-dyed bright pink frock, white "pinny" with bow at jaunty waist, and flaxen, fuzzy topping. She has to cross a 'bus route, and halts a moment consideringly. But she has done it many times before, and, anyway, every 'bus driver is a child's guardian too. At the shop pennies are held up. "Thope, Mithith." "If what, Polly?" asks the comfortable shopkeeper. The customer is deeply interested in a glass-fronted box of sugar biscuits, just at her eye level. But she rallies gallantly. "If—if—peath." And so home.

### Tom George Goes to School.

That cheerful building on the outer rim of the village is the school. "Magic casement" to our Tom George! See him at 9 a.m., head erect, march in time to the music:—

> Bobby Shafto's gone to sea,
> Silver buckles at his knee;
> When he comes back he'll marry me.
> Hurrah for Bobby Shafto!"

Tom George enters Paradise—a bright room, with lovely coloured pictures, plants and flowers, his own wee armchair with tray complete, modelling clay, coloured chalks and raffia to play at work with, and blackboards just his height all round the room, on which he is encouraged to write and draw by the gentle-voiced, smiling, lively young teacher. Say the mothers: "No bother getting the bairns to school! Very different to what it was in our young days." School! Council School Number 000 is much more to Tom George than Eton or Harrow to those other boys—from drabness to drill, singing, games, handwork, reading interesting books, sums concerning everyday affairs, nature study, talks about events in the great world away. Occasionally there is wireless. Tom George proposes to "speak America!"

### Wanted, Pithead Baths.

Ask bright-faced fourteen-year-old what he's going to do when he leaves school. "Go down the pit," is the reply; as who should say, "A magnificent career! What could be better?" One's twinge of regret is tempered by the thought that there are nine-and-sixty (more, maybe) ways of earning a living beside which coal-mining is as the toil of Sir Galahad. Only there should be brightness and beauty above ground, ample opportunity afforded those who go down to the bowels of the earth in cages to develop their talents and broaden their lives. This, of al places, cannot rest content with a school-leaving age of fourteen. There is more leisure, and means must be found to enrich it. Strangers in the land usually have two questions: "What is all that heavy thumping?" and "Why are there so many sweeps about?" We show them the heavy "poss stick," used by the mothers at their drudging task, the weekly wash—drudgery, indeed, when the men work at such a grimy job; and industrial and housing conditions make it almost impossible to keep house or child clean for an hour. Then we tell them that their "sweeps" are miners returning from work. Pithead baths are talked about. It is enormously important that this long looked-for reform should be hurried up. When the miner gets home the zinc bath is spread for him in front of the living-room fire, as it was a hundred years ago. It's the best he can do. But pithead baths are long overdue, and would be an immense boon to the man and his family. It has been often remarked that miners seldom or never wear overcoats going to or returning from work; sun or shower, balmy or biting blast, it is all the same. Most of us don't have two winter overcoats; let the miner leave all his dirt at the pit and come home snugly in his top coat, just like anybody else on winter days.

### The Cost of Bad Housing.

The cost of bad housing is frequently overlooked. The £15 houses afore noted have already cost thousands of pounds in sickness benefit and poor-law relief to the widows and children of men hurried to premature graves. Authority, treating tuberculosis, recently spent £200 on the occupants of one such house. Results to date:—Two graves, one cripple, still the old bad house. Total rebuilding is impracticable at present, but in addition to such new houses as have been and are to be built, much may be done to existing houses—roofs heightened, windows enlarged, extra rooms built on, water supply, sinks, wash-houses, bathrooms, water closets, street paving, and the knocking down of a few houses in a street's length to make a little public garden. It is so very well worth while, even if the houses last only another twenty years. Think of the mothers—and of the children! There is an oft-repeated gibe: "The people don't want bathrooms and will use them for coal storage." This argument always reminds me of a fragment of conversation overheard in a 'bus:—"I says to her, I says, I'm pleased to meet you, but you're no lady!" I shamelessly strained my ears in vain to catch the thread of so promising a story. What, conceivably, could be the frame of mind of a woman who thus greeted a new acquaintance? Well, what type of mind have folk who say the people won't use bathrooms? They already use telephones and wireless!

---

### DRINK, TOBACCO, AND LONGEVITY.

According to two American medical men, drinking and smoking conduce to longevity and to good health respectively. At every age, from 30 to 100, moderate drinkers, male or female, have a somewhat higher expectation of life than abstainers of the same age. Male heavy drinkers have a markedly lower expectation of life from 30 to 100 than moderate drinkers, and a markedly lower one than abstainers up to 60. Female heavy drinkers have a markedly lower one than either abstainers or moderates from 30 to 100. The "moderately steady" drinkers show consistently the highest expectation of life at all ages. Pipe smokers have an advantage in health over men who do not smoke. "I was in charge of over 500 soldiers at a post with marshes near by, and dysentery raged with virulence," says a surgeon who saw war service. "I noticed that the heaviest smokers, who went about with pipes in their mouths, did not contract the disease. I smoked all the time, and was free from it, so that smoking is in reality a great protective against disease."

## Figures and Tables

**Figure f1:**
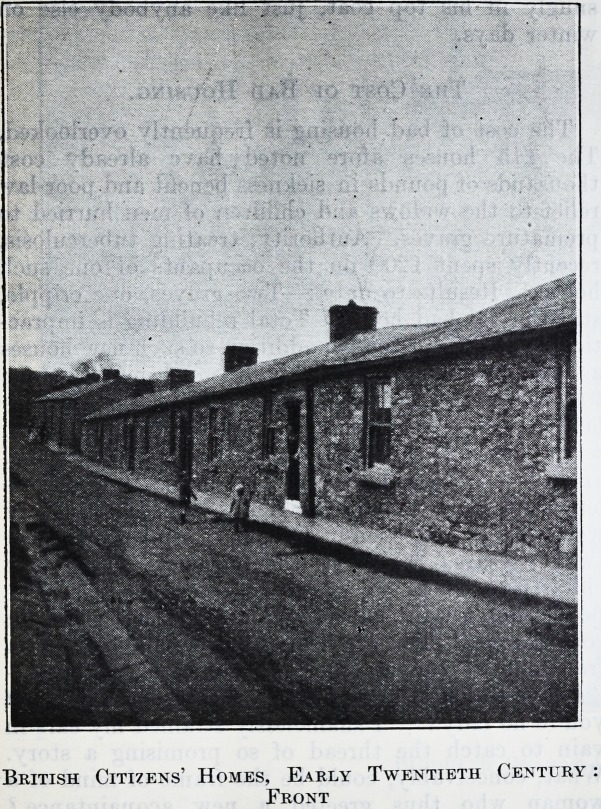


**Figure f2:**